# Whole-Transcriptome Sequencing Combined with High-Dimensional Proteomic Technologies Reveals the Potential Value of miR-135b-5p as a Biomarker for Hepatocellular Carcinoma

**DOI:** 10.1155/2023/6517963

**Published:** 2023-01-30

**Authors:** Yushan Zhang, Liangyu He, Lixin Pan, Chao Feng, Xi Wang, Yuting Tao, Tianyu Li, Qiuyan Wang, Hanif Ullah

**Affiliations:** ^1^Department of Biochemistry and Molecular Biology, School of Basic Medicine, Guangxi Medical University, Nanning, Guangxi 530021, China; ^2^Key Laboratory of Biological Molecular Medicine Research (Guangxi Medical University), Education Department of Guangxi Zhuang Autonomous Region, Nanning, Guangxi 530021, China; ^3^Center for Genomic and Personalized Medicine, Guangxi Medical University, Nanning 530021, China; ^4^Guangxi Key Laboratory for Genomic and Personalized Medicine, Guangxi Collaborative Innovation Center for Genomic and Personalized Medicine, Nanning 530021, China; ^5^Department of Urology, The First Affiliated Hospital of Guangxi Medical University, Nanning 530021, China

## Abstract

**Purpose:**

Hepatocellular carcinoma (HCC) is a disease with great heterogeneity and a high mortality rate. It is crucial to identify reliable biomarkers for diagnosis, prognosis, and treatment to improve clinical outcomes in patients with HCC. Alpha-fetoprotein (AFP) is not only a widely used biomarker in clinical practice but also plays a complicated role in HCC, and it has recently been considered to be related to immunotherapy. MicroRNAs (miRNAs) are regarded as key regulators and promising biomarkers of HCC. We investigated the role of an AFP-related miRNA, miR-135b-5p, in HCC progression.

**Methods:**

Identification of miR-135b-5p was performed based on a cohort of 65 HCC cases and the liver hepatocellular carcinoma cohort of The Cancer Genome Atlas (Asian people only). A combination of whole-transcriptome sequencing and high-dimensional proteomic technologies was used to study the role of miR-135b-5p in HCC.

**Results:**

Upregulation of miR-135b-5p was detected in patients with HCC with high serum AFP levels (AFP > 400 ng/ml). Elevated miR-135b-5p expression was associated with adverse prognosis. We also identified the relevance between high miR-135b-5p expression and tumor-related pathological characteristics, such as Edmondson grade and vascular invasion. We revealed tyrosine kinase nonreceptor 1 as a potential target of miR-135b-5p. Additionally, the transcriptional start site of miR-135b-5p had potential binding sites for SRY-box transcription factor 9, and the stemness properties of tumor cells were more remarkable in HCC with the upregulation of miR-135b-5p. The molecular characterization of the miR-135b-5p-high group was similar to that of the HCC subclasses containing moderately and poorly differentiated tumors. Finally, gene signatures associated with improved clinical outcomes in immune checkpoint inhibitor therapy were upregulated in the miR-135b-5p-high group.

**Conclusion:**

miR-135b-5p could be a biomarker for predicting the prognosis and antiprogrammed cell death protein 1 monotherapy response in HCC.

## 1. Introduction

Primary liver cancer is one of the most frequently occurring cancers, with a high mortality rate [1]. Hepatocellular carcinoma (HCC) accounts for approximately 75% of primary liver cancers and seriously threatens human health owing to its complicated etiology, insidious onset, and rapid development [2]. Despite the recent progress in diagnosis and therapy, the clinical outcomes of patients with HCC are not ideal [3, 4]. This may be due to the great tumor heterogeneity in HCC and the lack of reliable biomarkers for diagnosing, predicting prognosis, and monitoring response to treatment [5].

Alpha-fetoprotein (AFP) is a glycoprotein produced in early pregnancy, and its serum level decreases rapidly after birth, maintaining a low level throughout human life. High serum AFP levels can be detected in HCC patients, making it the most frequently used biomarker for surveillance and diagnosis [6]. Emerging evidence shows that AFP is not only a biomarker for diagnosis but also participates in the progression of HCC by regulating proliferation, apoptosis, and autophagy of tumor cells and the inhibition of immune cell function [7]. Recently, AFP was found to be associated with the response rate and/or survival to immune checkpoint inhibitor (ICI) therapy in patients with HCC [8, 9]. However, the performance of AFP as a diagnostic biomarker for HCC is controversial because of its unsatisfactory specificity and sensitivity, and the efficacy of AFP in prognostic prediction is influenced by cut-off criteria [10, 11]. Moreover, the capability of AFP in monitoring ICI therapy is not ideal, as objective remissions occurred regardless of AFP levels in combination with nivolumab and ipilimumab treatment [12]. Therefore, it is critical to identify reliable biomarkers of HCC.

By regulating the expression level of target genes, microRNAs (miRNAs) play vital roles in the cellular differentiation, tumor metabolic patterns, and tumor microenvironment (TME) of HCC [13–15]. Recent studies have suggested that miRNAs have the potential to serve as novel biomarkers or targets for HCC [16]. Additionally, AFP expression is regulated by miRNAs, indicating an association between miRNAs and AFP [17, 18]. Nevertheless, only a limited number of studies have explored the function of AFP-related miRNAs in HCC. Thus, the identification of AFP-related miRNAs may contribute to the development of HCC biomarkers.

This study is aimed at identifying AFP-related miRNAs and investigating an identified AFP-related miRNA, miR-135b-5p, by integrating whole-transcriptome sequencing, mass cytometry, and imaging mass cytometry. Thus, miR-135b-5p is considered as a promising biomarker for predicting the prognosis and antiprogrammed cell death protein 1 (anti-PD-1) therapy response in HCC.

## 2. Materials and Methods

### 2.1. Sample Collection

Sixty-five pairs of tissue samples including carcinoma (CA) and paired paracarcinoma (CP) were collected from patients with HCC who underwent surgical resection at the Affiliated Cancer Hospital of Guangxi Medical University from May 2018 to July 2019. Two experienced pathologists confirmed the diagnosis of HCC, and all participants had not received chemotherapy and radiotherapy before surgery. Paraffin histological sections were acquired from the pathology department of the Affiliated Cancer Hospital of Guangxi Medical University. The ethics committee of Guangxi Medical University approved the present study. Detailed pathological parameters and study cases are listed in Table 1 and Table S1.

### 2.2. Acquisition of miRNA Expression Profile

Total RNA isolation was conducted using TRIzol® reagent (Invitrogen) in accordance with the manufacturer's protocol. A NanoDrop (Thermo Scientific) and 2100 Bioanalyzer (Agilent) were used to perform RNA quantification and integrity analysis. Purification, reverse transcription, library construction, and sequencing were performed based on Illumina's instructions. The small RNA sequencing libraries were prepared using the Illumina TruSeq® Small RNA Library Preparation Kit. Quantification of the concentrations of the sequencing libraries was conducted via a Qubit 2.0 fluorometer dsDNA HS Assay (Thermo Scientific), and an Agilent Bioanalyzer 2100 (Agilent) was used to analyze the size distribution. Then, the libraries were used for cluster formation on an Illumina cBOT cluster generation system with HiSeq PE Cluster Kits (Illumina). Sequencing was performed using an Illumina HiSeq system following the protocols of Illumina. The miRNA expression profile was extracted from the small RNA expression profile. The miRNA Isoform Expression Quantification of Liver Cancer and clinical data were downloaded from The Cancer Genome Atlas (TCGA, https://portal.gdc.cancer.gov/). The Asian race (161 cases) was selected, and the corresponding miRNA expression profile was extracted; one sample with a missing survival time and status was excluded.

### 2.3. Quantitative Real-Time Polymerase Chain Reaction

The Mir-X miRNA First-Strand Synthesis Kit (Takara Bio), Mir-X miRNA First-Strand Synthesis Kit (Takara Bio), and LightCycler® 96 Instrument (Roche, USA) were applied to perform reverse transcription and quantitative real-time polymerase chain reaction (qRT-PCR), according to the manufacturers' instructions. The primer sequence of miR-135b-5p was 5′-GGAGGCTTTTCATTCCTATGTGA-3′, and the primer was synthesized by Takara Biomedical Technology Co., Ltd. Calculation of relative changes in expression was based on the 2^−*Δ*CT^ method, and all reactions were performed in triplicate.

### 2.4. Acquisition of mRNA Expression Profile

The extraction of total RNA was the same as that in the previous description. RNA purification, library construction, and paired-end sequencing were conducted based on Illumina's instructions. FastQC and fastp were used for data preprocessing, including raw sequencing data quality control, adapter trimming, and quality filtering. Clean data mapping, RNA sequence assembly, and data merging were conducted using HISAT2, StringTie, and Cufflinks.

### 2.5. Differentially Expressed Gene and Pathway Enrichment Analyses

DESeq2 was applied to identify differentially expressed miRNAs (DEmiRNAs) and differentially expressed genes (DEGs) with cut-off criteria of an adjusted *p* value < 0.01 and |log_2_(fold change)| > 1. DAVID (https://david.ncifcrf.gov/) and KOBAS (http://kobas.cbi.pku.edu.cn/) platforms were used to perform Gene Ontology (GO) analysis and Kyoto Encyclopedia of Genes and Genomes (KEGG) analysis, respectively. *p* value < 0.05 was regarded as significant.

### 2.6. Prediction of Target Genes and Transcription Factors (TFs)

In total, 2670 potential target genes were discovered by integrating the results of miRDB (http://www.mirdb.org/), miRwalk (http://mirwalk.umm.uni-heidelberg.de/), and TargetScan (https://www.targetscan.org/). The TransmiR v2.0 database contains information about TF-miRNA regulation, which was used to predict interactions [19].

### 2.7. Gene Set Enrichment Analysis (GSEA)

GSEA 4.1.0 was used to conduct GSEA based on mRNA expression profiles. The parameters were set as the defaults. An absolute value of the normalized enrichment score ≥ 1 and *p* value < 0.05 were regarded as criteria for statistical significance.

### 2.8. Mass Cytometry

Single-cell suspension preparation, antibody conjugation, cell staining, and data acquisition for CyTOF were performed as previously described [20]. The antibody panel is listed in Table S4. FCS files of 48 CA samples (20 samples with high miR-135b-5p expression and 28 samples with low miR-135b-5p expression) were then concatenated, normalized, and debarcoded. Five thousand living cells per sample were randomly extracted and analyzed using the R package cytofkit, and cells were clustered with FlowSOM [21, 22]. Distributed stochastic neighbor embedding (t-SNE) was used to conduct dimensionality reduction to realize visualization [23]. A heatmap was plotted based on the marker expression of each cell cluster (after normalization). Comparisons of the marker expression between the miR-135b-5p-high and miR-135b-5p-low groups were performed with a Wilcox test.

### 2.9. Imaging Mass Cytometry

Paraffin histological sections (28 samples with high miR-135b-5p expression and 19 samples with low miR-135b-5p expression) were incubated at 65°C for 2 h in an oven and then were deparaffinized by incubating them with xylene. Sequential rehydration was carried out from absolute ethanol to 75% ethanol. Tris-EDTA buffer was used for antigen retrieval. Sections were then washed with PBS and blocked in DPBS containing 3% BSA at room temperature for 45 mins. Sections were transferred to a humid chamber and incubated with an antibody cocktail conjugated with metal at 4°C overnight. Sections were counterstained with Cell-IDTM Intercalator-Ir (Fluidigm) in DPBS at room temperature for 30 mins and then washed with DPBS containing 0.1% Triton-X (Thermo Scientific) and absolute DPBS. Sections were dried at room temperature. A Hyperion imaging mass cytometer (Fluidigm) was used for data acquisition at a frequency of 200 Hz and a resolution of 1 *μ*m. In total, 418 regions of interest (ROIs; from 5 to 13 ROIs per section) were detected, and the area of the ROI was 1 × 1 mm^2^. Data obtained via a Hyperion imaging mass cytometer were exported as tiff files using MCD viewer. CellProfiler 3.1.9 was used to generate cell segmentation masks and extract the intensity of markers in the panel based on the tiff files (Table S5) [24]. The tiff files with cell masks were inputted into histoCAT 1.73 for analyses [25]. Markers within the tumor region of each ROI were quantified based on an in-house method.

### 2.10. Hematoxylin and Eosin Staining

Deparaffinization and rehydration were performed as previously described. A PAP pen was used to draw lines around the tissue, and hematoxylin was added to the sections to cover the tissue area. Sections were incubated at room temperature for 5 mins. Then, sections were washed for 3 mins and immersed in the distilled H_2_O. Next, 1% hydrochloric acid alcohol was added to the sections, and the sections were washed with distilled H_2_O for 3 mins after differentiation for a few seconds. The eosin staining solution was added to the sections to cover the tissue area, and sections were then incubated at room temperature for 2 mins. Sections were washed for 3 mins and immersed in distilled H_2_O. For dehydration, sections were immersed in absolute ethanol for 2 mins and xylene for 5 mins (twice). Sections were dried and sealed with neutral gum before observations.

### 2.11. Single Sample Gene Set Enrichment Analysis (ssGSEA)

The ssGSEA was performed to calculate the enrichment scores of seven immune pathways,eleven immune cell-related gene sets, and three gene signatures associated with anti-PD-1 therapy [26–28].

### 2.12. Statistical Analysis

An unpaired *t*-test and Mann-Whitney test were chosen for statistical analysis. An evaluation of the correlation between indicated genes was performed via the Pearson correlation coefficient analysis. Survival curves were estimated by performing the Kaplan-Meier analysis, whereas the comparisons between indicated groups were conducted using the log-rank test. Risk factors affecting overall survival (OS) were evaluated by performing the Cox regression analysis. The association between miR-135b-5p and clinicopathological features was evaluated by performing a chi-squared test. These analyses were conducted using GraphPad Prism 7 and SPSS 24, and a *p* value < 0.05 was considered statistically significant.

## 3. Results

### 3.1. miR-135b-5p Was Identified as an AFP-Related miRNA

**Figure 1:**
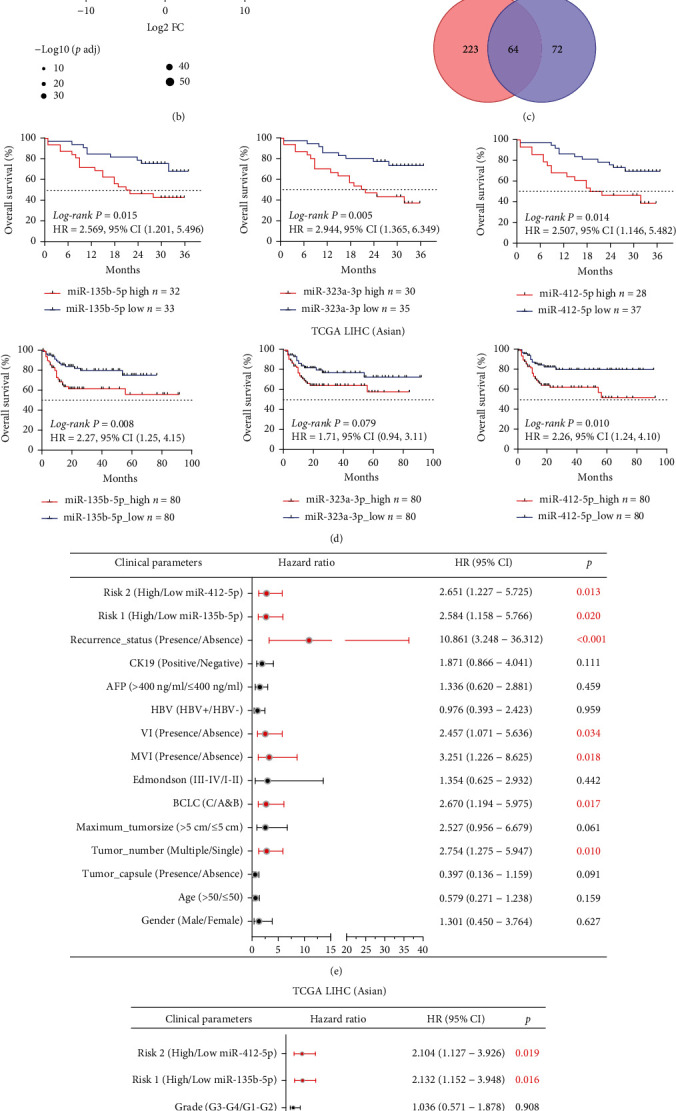
The expression of miR-135b-5p in HCC and its association with prognosis. (a) Volcano plot shows DEmiRNAs between the AFP-high and AFP-low groups. (b) Volcano plot shows DEmiRNAs between the CA and CP groups. (c) Venn diagram shows the number of common DEmiRNAs. (d) The Kaplan-Meier curves show the OS of patients, characterized by low or high expression of indicated miRNA (based on the median expression level of each miRNA). (e) Forest plot shows the risk factors evaluated by the univariate Cox proportional hazard regression model. (f) Forest plots show the risk factors evaluated by the univariate (top) or multivariate (bottom) Cox proportional hazard regression model. (g) qRT-PCR quantifies the expression of miR-135b-5p in the AFP-high and AFP-low groups. ^∗^*p* < 0.05.

Sixty-five patients with HCC were divided into two groups based on their preoperative serum AFP levels. We set AFP > 400 ng/ml as the cut-off criterion, as it is considered to be associated with adverse outcomes and oncological characteristics of HCC [29]. There were 22 cases in the AFP-high group (serum AFP > 400 ng/ml) and 43 in the AFP-low group (serum AFP ≤ 400 ng/ml). We identified DEmiRNAs between the AFP-high and AFP-low groups as well as those between the CA and CP groups. A total of 136 and 287 DEmiRNAs were identified between the AFP-high and AFP-low groups and the CA and CP groups, respectively (Figures 1(a) and 1(b)).

Following integrated analysis, 64 DEmiRNAs were shown in the Venn diagram (Figure 1(c)).

It includes 59 consistently upregulated miRNAs and 3 consistently downregulated miRNAs in CA and AFP-high groups (Table S2). We assessed the influence of these 62 DEmiRNAs on prognosis in our cohort and the Asian liver hepatocellular carcinoma (LIHC) patient cohort of The Cancer Genome Atlas (TCGA).

As shown in Figure 1(d), these two miRNAs were associated with patient survival. Moreover, Cox regression analyses showed that miR-135b-5p is a risk factor for overall survival (OS) in patients. The relationship between miR-135b-5p and clinicopathological features was determined, and elevated miR-135b-5p expression was found to be related to age, CK19 expression, Edmondson grade, tumor number, and vascular invasion (Figure S1(a)). We quantified miR-135b-5p expression in 19 HCC samples by qRT-PCR. As expected, miR-135b-5p was upregulated in the AFP-high group (Figure 1(g)). The efficacy of AFP in prognostic prediction using different cut-off criteria was examined, and the results demonstrated that miR-135b-5p had a better performance in prognostic prediction (Figure S1(b)), as the OS of patients in the AFP-high and AFP-low groups showed no significant difference. These results indicate that miR-135b-5p is highly expressed in HCC cases with high serum AFP levels, and high miR-135b-5p expression is related to poor prognosis. We consider that miR-135b-5p as an AFP-related miRNA could be a promising biomarker for HCC.

### 3.2. Study of the Potential Regulatory Mechanism of miR-135b-5p via Whole-Transcriptome Sequencing

**Figure 2:**
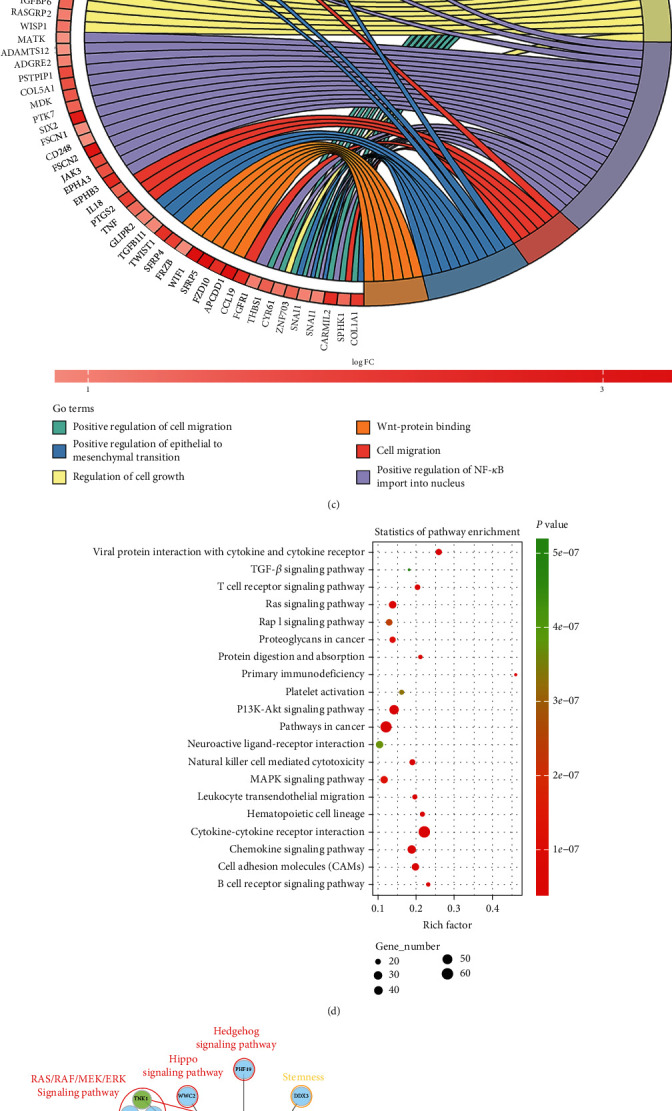
Differentially expressed gene analysis, pathway enrichment analysis, and miRNA-related bioinformatics analyses. (a) Heatmap shows DEGs between the miR-135b-5p-high and miR-135b-5p-low groups. (b) Heatmap shows indicated DEGs between the miR-135b-5p-high and miR-135b-5p-low groups. (c) Chordal graph shows certain GO pathways. (d) Dot plot shows the top 20 KEGG pathways (ranked by the *p* value). (e) Regulatory network between miR-135b-5p and potential target genes (left); only the tumor-promoting genes are shown based on previously published research; scatter plot shows the correlation between miR-135b-5p and TNK1 (right). (f) Regulatory network between miR-135b-5p and coexpressed genes (left); red lines and blue lines indicate positive correlation and negative correlation, respectively; scatter plot shows the correlation between miR-135b-5p and SOX9 (right). (g) Box plot shows the expression of LCSC-related genes in the miR-135b-5p-high and miR-135b-5p-low groups.

We performed a differential gene expression analysis to determine the potential regulatory mechanism of miR-135b-5p in HCC. A total of 1491 DEGs were identified, including 1254 upregulated and 237 downregulated DEGs in the miR-135b-5p-high group (Figure 2(a)). Liver cancer stem cells (LCSCs), differentiation-related genes, and epithelial-mesenchymal transition- (EMT-) induced transcription factors were highly expressed in the miR-135b-5p-high group (Figure 2(b)) [30–32]. GO and KEGG analyses were conducted to clarify the pathways that were enriched by upregulated genes. GO analysis revealed positive regulation of cell migration, EMT, and NF-*κ*B import into nuclear signaling pathways (Figure 2(c)). The MAPK, PI3K/AKT, Ras, and TGF-*β* signaling pathways were enriched in KEGG analysis (Figure 2(d)). miRNAs play a role in the posttranscriptional suppression of mRNA translation or facilitation of mRNA degradation [33]. Thus, three platforms were used to predict the targets of miR-135b-5p, and a regulatory network was established using Cytoscape (Figure 2(e)). TNK1, an inhibitor of the MAPK/ERK signaling pathway, was also identified [34]. This result indicates that miR-135b-5p is likely to promote HCC malignancy by activating the MAPK/ERK signaling pathway. Genes coexpressed with miR-135b-5p were identified using Pearson's correlation coefficient analysis for further study, and the regulatory network was established using Cytoscape (Figure 2(f)). A transcription factor that maintains the self-renewal and tumorigenicity of LCSCs, SOX9, was found to be positively correlated with miR-135b-5p (Figure 2(f)) [35]. As various interaction patterns between transcription factors and miRNAs have been proposed, the relationship between SOX9 and miR-135b-5p was evaluated using the TransmiR database [19, 36]. The predicted results indicated that the transcriptional start site of miR-135b-5p had potential binding sites for SOX9, suggesting that SOX9 may increase the expression of miR-135b-5p by binding to its promoter (Table S3). As the MAPK/ERK signaling pathway and SOX9 mediated the differentiation and cell fate in HCC, respectively, we compared the expression of genes encoding putative biomarkers for LCSCs between the miR-135b-5p-high and miR-135b-5p-low groups [30, 35, 37]. These genes were highly expressed in the miR-135b-5p-high group (Figure 2(g)). Taken together, we assumed that miR-135b-5p activates the MAPK/ERK signaling pathway by targeting TNK1, while miR-135b-5p expression is potentially regulated by SOX9. Moreover, the relatively higher LCSC-related gene expression levels suggest a potential association between tumor stemness properties and miR-135b-5p.

### 3.3. Analyses Based on High-Dimensional Proteomic Technologies Identify Remarkable Stemness Properties in HCC with High miR-135b-5p Expression

**Figure 3:**
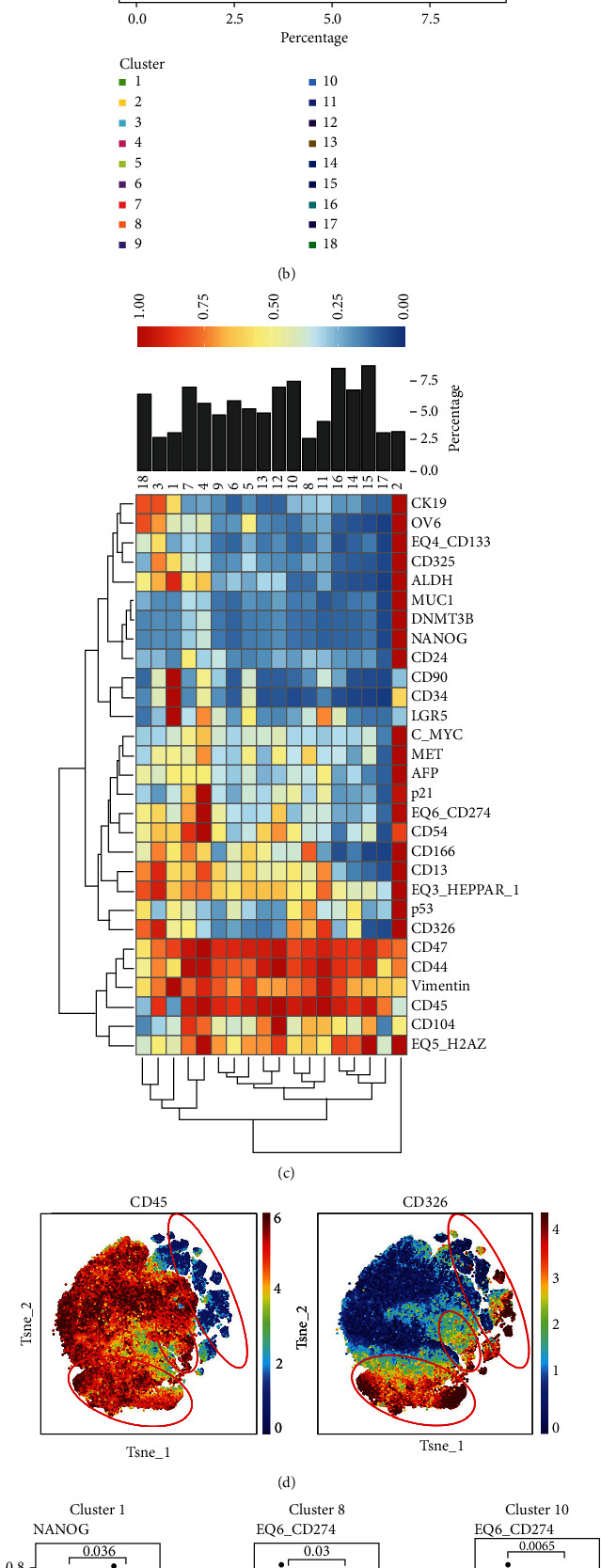
The TME landscape of HCC and characteristics of specific clusters. (a) t-SNE map shows the TME landscape of HCC. (b) Bar plot shows the percentages of the 18 cell clusters. (c) Heatmap shows the expression of 29 cellular markers of the 18 cell clusters. (d) t-SNE maps show the landscape of CD45 and CD326; CD45-/lowCD326+ and CD45+CD326+ cell clusters were labeled by red circles. (e) Box plots show the differentially expressed markers between the miR-135b-5p-high and miR-135b-5p-low groups within indicated cell clusters; numbers in the box plots indicate the *p* value.

**Figure 4:**
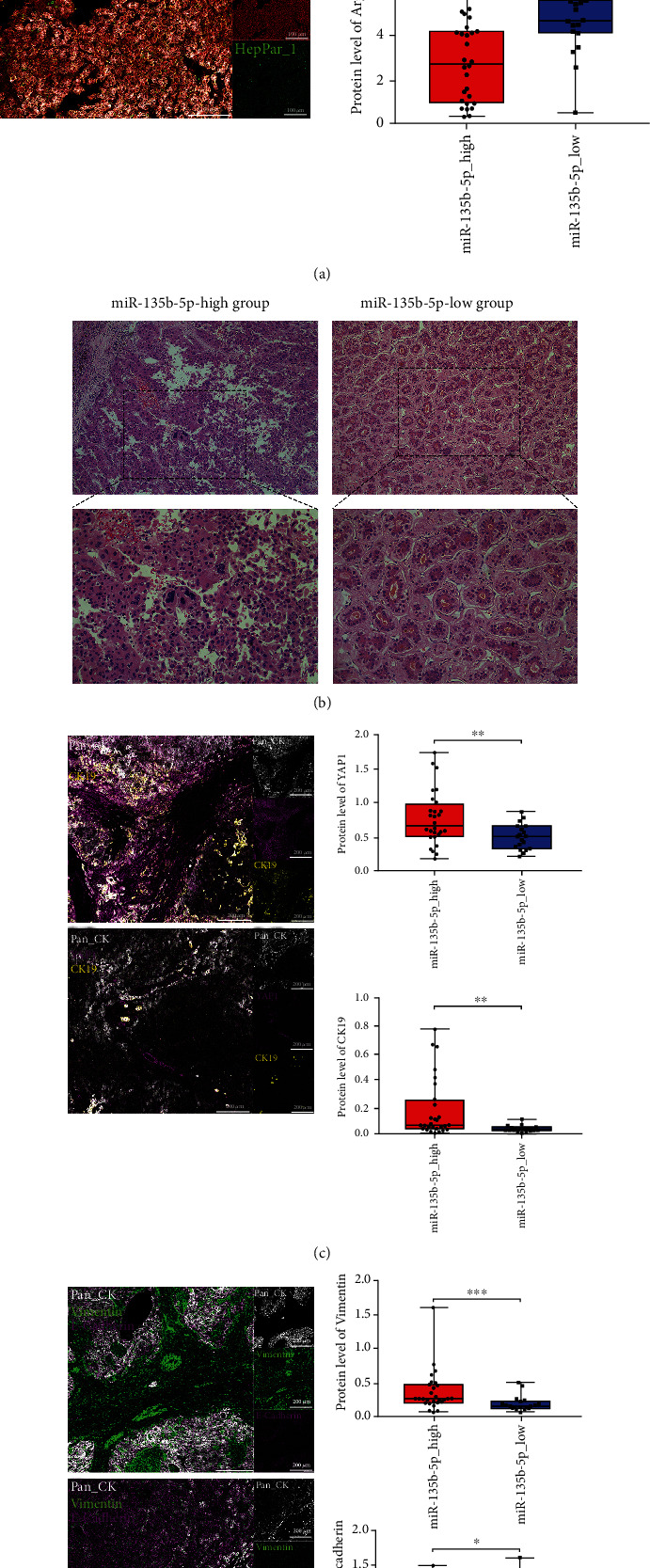
Distinct tumor differentiation extent between the miR-135b-5p-high and miR-135b-5p-low groups. (a) Representative IMC images show the expression of hepatocyte-specific markers in miR-135b-5p-high (top left) and miR-135b-5p-low groups (bottom left); box plots show the expression of hepatocyte-specific markers in the miR-135b-5p-high and miR-135b-5p-low groups (right). (b) Images of hematoxylin and eosin staining show differentiated degree of tumor cells in the miR-135b-5p-high and miR-135b-5p-low groups, exhibited by different magnification (top: ×100 and bottom: ×200). (c) Representative IMC images show the expression of YAP1 and CK19 in the miR-135b-5p-high (top left) and miR-135b-5p-low groups (bottom left); box plots show the expression of YAP1 and CK19 in the miR-135b-5p-high and miR-135b-5p-low groups (right). (d) Representative IMC images show the expression of EMT-related markers in the miR-135b-5p-high (top left) and miR-135b-5p-low groups (bottom left); box plots show the expression of EMT-related markers in the miR-135b-5p-high and miR-135b-5p-low groups (right). ^∗^*p* < 0.05, ^∗∗^*p* < 0.01, and ^∗∗∗^*p* < 0.001.

Considering that LCSCs contribute to the recurrence and treatment failure of HCC, we further validated the potential association between tumor stemness properties and miR-135b-5p at the proteomic level, as the protein is the executor of physiological functions [38]. Therefore, CyTOF, a multivariate single-cell proteomic technique, was used [39]. The t-SNE algorithm was used to visualize the phenotypic diversity of living cells in the miR-135b-5p-high and miR-135b-5p-low groups, and 18 cell clusters were identified using the FlowSOM algorithm (Figures 3(a) and 3(b)). Clusters 1, 2, and 18 (CD45-/lowCD326+) were defined as stem-like cell clusters owing to the expression of CD326, whereas clusters 3, 7, 8, 10, 11, and 14 (CD45+CD326+) were represented as malignant cell clusters (Figures 3(c) and 3(d)) [40]. The expression levels of 29 markers in these clusters were compared between the miR-135b-5p-high and miR-135b-5p-low groups. NANOG was highly expressed in the miR-135b-5p-high group within clusters 1 and 14, and elevated expression of C-MYC and CD24 was observed in the miR-135b-5p-high group within cluster 14, indicating a more remarkable stemness property of tumor cells in HCC with high miR-135b-5p expression (Figure 3(e)) [30]. Moreover, one of the immune checkpoints, programmed death ligand 1 (PD-L1), showed relatively high expression in the miR-135b-5p-high group within clusters 8 and 10 (Figure 3(e)) [41]. Another proteomic technique, IMC, was used to further compare the stemness properties of tumor cells between the miR-135b-5p-high and miR-135b-5p-low groups [42]. As hepatocyte-specific markers, arginase and HepPar1 were downregulated in the tumor cells within the miR-135b-5p-high group, suggesting a dedifferentiation of tumor cells in this group (Figure 4(a)) [43]. Hematoxylin and eosin staining experiments were conducted to verify the differences in tumor cell differentiation between the miR-135b-5p-high and miR-135b-5p-low groups. As shown in the representative images, tumor giant cells and mitosis were observed in the miR-135b-5p-high group, whereas tumor cells in the miR-135b-5p-low group showed a better degree of differentiation (Figure 4(b)). YAP1, a crucial member of the Hippo signaling pathway, and CK19, a marker of LCSCs, were highly expressed in the miR-135b-5p-high group, indicating that the Hippo signaling pathway was dysregulated and the stemness properties of tumor cells were more remarkable (Figure 4(c)) [30, 44]. EMT is a crucial biological process closely related to the stemness of HCC [45]. Thus, the expression of EMT-related markers was compared between the two groups, and we found that vimentin and E-cadherin were highly expressed in the miR-135b-5p-high and miR-135b-5p-low groups, respectively (Figure 4(d)). These results demonstrated that the stemness properties of tumor cells were more remarkable in HCC with high miR-135b-5p expression, which validated our transcriptome findings.

### 3.4. miR-135b-5p Is Associated with Tumor Dedifferentiation

Tumor differentiation is closely related to the stemness of the tumor cells [46]. Consequently, we used the gene signature of S1–S3 subclasses with distinct features of cellular differentiation and molecular pathways to examine the relationship between miR-135b-5p and tumor dedifferentiation [47]. The gene expression of the miR-135b-5p-high group was similar to that of the S1 and S2 subclasses, which were composed of moderately and poorly differentiated HCC (Figure S2(a)). Hepatocyte function-related genes showed relatively high expression in the miR-135b-5p-low group, reflecting the better-differentiated signature of this group (Figure S2(b)). Additionally, the results of GSEA showed that EMT, MYC targets, PI3K/AKT/mTOR, and TGF-*β* signaling pathway gene sets were enriched in the miR-135b-5p-high group, whereas metabolism-related gene sets were enriched in the miR-135b-5p-low group (Figures S2(c) and 2(d)). These results further suggest that elevated miR-135b-5p expression is related to poor HCC differentiation.

### 3.5. miR-135b-5p Is a Potential Biomarker for Anti-PD-1 Therapy Response

**Figure 5:**
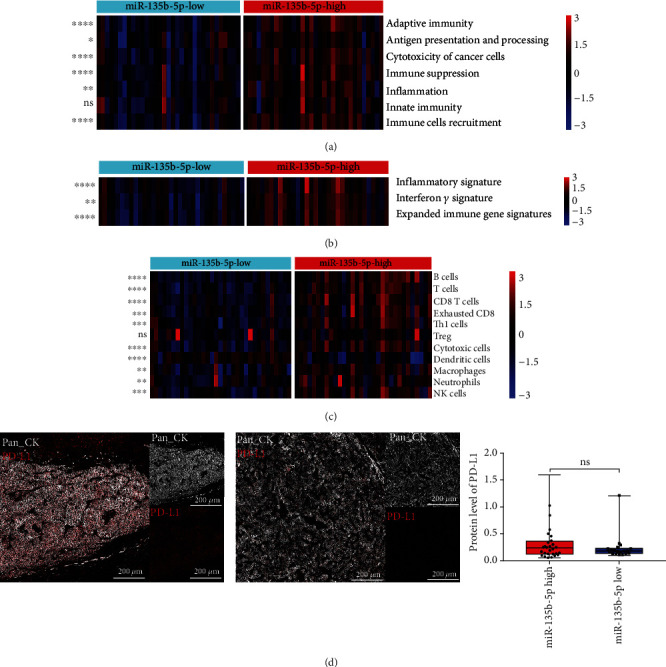
Differential expression of immune pathways, immune cells, and ICI therapy-related gene signatures between the miR-135b-5p-high and miR-135b-5p-low groups. (a) Heatmap shows the expression of seven immune pathway-related gene signatures in the miR-135b-5p-high and miR-135b-5p-low groups. (b) Heatmap shows the expression of 11 immune cell-related gene signatures in the miR-135b-5p-high and miR-135b-5p-low groups. (c) Heatmap shows the expression of three anti-PD-1 therapy-related gene signatures in the miR-135b-5p-high and miR-135b-5p-low groups. (d) Representative IMC images show the expression of PD-L1 in the miR-135b-5p-high (left) and miR-135b-5p-low groups (middle); box plot shows the expression of PD-L1 in the miR-135b-5p-high and miR-135b-5p-low groups (right). ^∗^*p* < 0.05, ^∗∗^*p* < 0.01, ^∗∗∗^*p* < 0.001, and ^∗∗∗∗^*p* < 0.0001. ns: no significance.

Abnormal PD-L1 expression within the malignant cell clusters in the miR-135b-5p-high group, identified by CyTOF, indicated a different immune signature between the miR-135b-5p-high and miR-135b-5p-low groups. Therefore, we selected and compared the enrichment of seven immune pathway-related gene signatures and 11 immune cell gene sets between the two groups using the ssGSEA algorithm [26]. Upregulation of adaptive immunity, antigen processing and presentation, cytotoxicity of cancer cells, immune suppression, inflammation, and immune cell recruitment-related gene signatures were observed in the miR-135b-5p-high group (Figure 5(a)). Gene signatures related to B cells, T cells, CD8 T cells, exhausted CD8 T cells, T helper 1 cells, Tregs, cytotoxic cells, macrophages, neutrophils, and natural killer cells were also enriched in the miR-135b-5p-high group (Figure 5(b)). We further evaluated the expression of gene signatures associated with survival and/or response to anti-PD-1 therapy in the miR-135b-5p-high and miR-135b-5p-low groups based on the ssGSEA algorithm [27, 28]. Sangro inflammatory signature, interferon gamma signature, and expanded immune gene signature were upregulated in the miR-135b-5p-high group (Figure 5(c)). As high PD-L1 expression has been reported to correlate with better anti-PD-1 therapy response or OS, we compared PD-L1 expression in tumor cells between the miR-135b-5p-high and miR-135b-5p-low groups [48]. Distinct PD-L1 expression between the two groups was observed in representative IMC images, with higher PD-L1 expression in the tumor cells of the miR-135b-5p-high group (Figure 5(d)). These results indicated that the miR-135b-5p-high group was characterized by high immune infiltration, with activation of immune pathways, and HCC patients with high miR-135b-5p expression were more likely to derive benefits from anti-PD-1 therapy compared to those with low miR-135b-5p expression. Therefore, miR-135b-5p may be a potential biomarker for anti-PD-1 therapy response of HCC.

## 4. Discussion

HCC is a malignant tumor with high heterogeneity, and the identification of reliable biomarkers has become a consensus to improve patient outcomes [5]. The detection of AFP is an effective strategy for HCC surveillance and diagnosis, and high AFP levels are associated with worse clinicopathological and molecular characteristics [49]. miRNAs, as regulators of mRNAs, participate in various HCC processes and are also considered valuable biomarkers for diagnosis, prognosis, and treatment [16]. The identification of AFP-related miRNAs may be conducive to the discovery of novel biomarkers and the improvement of clinical outcomes.

The upregulation of miR-135b-5p in HCC with elevated AFP levels was verified using qRT-PCR. miR-135b-5p was reported to promote tumor progression in colorectal, gastric, and pancreatic cancers [50–52]. However, another study demonstrated that miR-135b-5p acts as a suppressor in HCC by inhibiting androgen receptor expression [53]. Androgen receptors appear to be differentially expressed in HCC [54]. Thus, the role of miR-135b-5p in HCC requires further investigation.

We conducted differential gene expression and pathway enrichment analyses for a preliminary investigation. LCSCs, differentiation-related genes, and EMT-induced transcription factors were highly expressed in HCC cells with high miR-135b-5p expression levels. The enrichment of tumor-related signaling pathways suggests that miR-135b-5p is involved in several biological processes. Specifically, by targeting TNK1, an inhibitor of the Grb2-Sos1 GEF complex, miR-135b-5p may activate the MAPK/ERK signaling pathway and in turn promote tumor progression [34]. Moreover, we found that a member of the SRY-box family that functions as a regulator of cell fate determinations, SOX9, was positively related to miR-135b-5p [55]. Based on the results of the TransmiR database, we assumed that SOX9 binds to the promoter and regulates miR-135b-5p expression. Genes encoding putative biomarkers for LCSCs were highly expressed in the miR-135b-5p-high group, suggesting that miR-135b-5p is related to the stemness of tumor cells [30].

We further verified our hypothesis using CyTOF and IMC. CyTOF can be seen as a continuation of developments in fluorescence-based cytometry, which overcomes the limited detection parameters and interference from spectral overlap, allowing the simultaneous analysis of dozens of cellular markers in individual cells [39]. Using CyTOF, we revealed different marker expression patterns of CD45-/lowCD326+ stem-like cells and CD45+CD326+ malignant cells between the miR-135b-5p-high and miR-135b-5p-low groups. Compared to the miR-135b-5p-low group, higher expression of CD24, C-MYC, and NANOG was observed in the miR-135b-5p-high group within stem-like or malignant cell clusters, suggesting more remarkable stemness properties of tumor cells in this group. Interestingly, cluster 14 showed a high expression of both CD24 and NANOG. The association between cluster 14 and miR-135b-5p may require further exploration, as CD24 can promote NANOG expression via phosphorylation of signal transducer and activator of transcription 3 [56]. IMC is an expansion of mass cytometry that can detect dozens of proteins in each ROI of paraffin-embedded tissue sections by combining laser ablation with mass spectrometry [42]. The results of IMC suggested a link between miR-135b-5p and dedifferentiation of tumors, reflected by the high expression of arginase and HepPar1 in tumor cells of the miR-135b-5p-high group [43]. Poor tumor differentiation in the miR-135b-5p-high group was verified using hematoxylin and eosin staining. Consistent with the transcriptome observations, CK19 was highly expressed in the miR-135b-5p-high group. YAP1 is a key member of the Hippo signaling pathway and a transcriptional coactivator regulating tumor cell proliferation, cell cycle, survival, and differentiation in combination with TEAD and ultimately leading to the occurrence and progression of HCC [44]. The Hippo signaling pathway might be dysregulated in the miR-135b-5p-high group because of the high expression of YAP1, which might be related to the poor differentiation of tumor cells in this group. Poorly differentiated tumors tend to shift to the mesenchymal state but not to the epithelial state [57]. Upregulation of vimentin and downregulation of E-cadherin indicated that the tumor cells in the miR-135b-5p-high group possessed a mesenchymal phenotype.

Tumor dedifferentiation is defined as the loss of cell identity and the reacquisition of stem cell features, which induces tumor progression and therapy resistance [46]. The potential link between miR-135b-5p and dedifferentiation was further investigated after observing the upregulation of LCSC-related markers in the miR-135b-5p high-expression group. The S1–S3 molecular subclasses are a consensus classification established based on 603 HCC cases [47]. Specifically, subclasses S1 and S2 were composed of moderately and poorly differentiated tumors, respectively, while subclass S3 included well-differentiated tumors. The Wnt and TGF-*β* signaling pathways were activated in the S1 subclass, S2 was characterized by MYC and AKT activation as well as high proliferation, and S3 was related to differentiation. Our results showed that the signature genes of the S1 and S2 subclasses were highly expressed in the miR-135b-5p-high group, whereas the genes that were highly expressed in the well-differentiated S3 subclass were highly expressed in the miR-135b-5p-low group. Moreover, gene sets enriched in subclasses S1, S2, and S3 were also activated in the miR-135b-5p-high and miR-135b-5p-low groups. The similarity of the molecular characterization between the miR-135b-5p-high group and the subclasses S1 and S2 demonstrated that elevated miR-135b-5p expression was related to poor HCC differentiation.

By modifying immune status, immunotherapy has been used to complement conventional cancer treatments, which have recently played an important role in oncology. As one of the immunotherapy strategies, ICI therapies, including anti-PD-1 and PD-L1 therapy, are considered to be a revolutionary milestone, disturbing coinhibitory signaling pathways, activating antitumor immune responses, and leading to the eradication of tumor cells [58]. However, only a percentage of the patients responded to ICI therapy. One of the determinants of anti-PD-1 therapy is the high expression of PD-L1 in tumor cells [59]. Analysis of CyTOF showed relatively higher PD-L1 expression in clusters 8 and 10 (CD45+CD326+) in the miR-135b-5p-high group compared to the miR-135b-5p-low group. Immune infiltration, especially of CD8 T cells and natural killer cells, is considered to be related to a better response to anti-PD-1 therapy [60]. As a higher expression of immune cells and immune pathway-related gene signatures was observed in the miR-135b-5p-high group, we hypothesized that the patients in this group might be more adapted to anti-PD-1 therapy. Previous studies have identified gene signatures associated with improved clinical outcomes in anti-PD-1 monotherapy, including antigen presentation, cytotoxicity, immune exhaustion/checkpoints, and inflammatory and interferon gamma-related genes [27, 28]. These gene signatures were upregulated in the miR-135b-5p-high group.

Although the efficacy of AFP as a screening, diagnostic, and prognostic marker for HCC is not ideal, it is still the most frequently used biomarker in the management of HCC [61]. In this study, we found that miR-135b-5p is an independent prognostic factor of HCC, which is related to AFP high expression HCC group. Further transcriptomic and proteomic analyses showed that miR-135b-5p is closely associated with tumor stemness properties, similar to the molecular characteristics of patients with HCC with high AFP levels. Notably, we found that the specific cell cluster with high expression of PD-L1 is enriched in miR-135b-5p-high HCC group, suggesting that miR-135b-5p may be used as a marker for screening immunotherapy-adaptive population. Our findings provide a theoretical basis and potential targets for the development of a new AFP-related biomarker for the prognosis and therapeutic evaluation of HCC.

Our study has some limitations. First, we identified miR-135b-5p in a cohort of only 65 cases; thus, a larger and more independent cohort should be used to verify the results. Second, the interactions between miR-135b-5p, TNK1, and SOX9 require further verification via luciferase reporter and chromatin immunoprecipitation assays. Third, more in vitro and in vivo experiments are necessary to evaluate the effects of miR-135b-5p on phenotypic changes in HCC.

## 5. Conclusion

Overall, by integrating whole-transcriptome sequencing, CyTOF, and IMC, we revealed the role of an AFP-related miRNA, miR-135b-5p, in HCC. Elevated miR-135b-5p expression was related to poor prognosis and tumor-related pathological characteristics. miR-135b-5p might activate the MAPK/ERK signaling pathway by targeting TNK1, and its expression was probably regulated by SOX9. miR-135b-5p promoted the stemness properties of tumor cells and related to tumor dedifferentiation. Moreover, HCC with high miR-135b-5p expression was more likely to derive benefits in anti-PD-1 therapy. We suggested that miR-135b-5p is a potential biomarker for predicting prognosis and anti-PD-1 therapy response in HCC.

## Figures and Tables

**Table 1:** Comparison of clinical pathological parameters between patients in the AFP-high and AFP-low groups.

Parameter	Case	AFP high	AFP low	*p* value	*x* ^2^
	65	22	43		
Age (year)					
≤50	29	13	16	0.093	5.56
>50	36	9	27		
BCLC					
A/B	51	16	35	0.421	3.52
C	14	6	8		
CK19					
Positive	19	12	7	0.001	17.40
Negative	46	10	36		
Edmondson stage					
I-II	32	6	26	0.006	5.72
III-IV	31	16	15		
HBV					
HBV +	51	18	33	0.638	0.29
HBV -	14	4	10		
Maximum tumor size (cm)					
≤5	21	4	17	0.082	0.12
>5	44	18	26		
Microvascular invasion					
Presence	41	16	25	0.158	1.25
Absence	23	5	18		
Recurrence					
Presence	35	12	23	0.936	3.52
Absence	30	10	20		
Gender					
Male	54	16	38	0.111	0.88
Female	11	6	5		
Tumor capsule					
Presence	59	20	39	0.783	0.29
Absence	5	2	3		
Tumor number					
Single	26	15	11	0.239	4.52
Multiple	16	13	3		
Vascular invasion					
Presence	13	6	7	0.294	4.99
Absence	52	16	36		

HBV: hepatitis B virus.

## Data Availability

The data that support the findings of this study are available from the corresponding authors upon reasonable request.

## Supplementary Materials

Supplementary Materials Figure S1: the efficacy of AFP for prognostic prediction in patients with HCC. Figure S2: the differences in molecular characterization between the miR-135b-5p-high and miR-135b-5p-low groups. Table S1: study cases. Table S2: 59 consistently upregulated miRNAs and 3 consistently downregulated miRNAs in CA and AFP-high group. Table S3: prediction result of TransmiR database. Table S4: antibody panel of CyTOF. Table S5: antibody panel of IMC.
