# Supplementary material for: Whole-Transcriptome Sequencing Combined with High-Dimensional Proteomic Technologies Reveals the Potential Value of miR-135b-5p as a Biomarker for Hepatocellular Carcinoma
Source: Biomed Res Int. 2023 Jan 30;2023:6517963. doi: 10.1155/2023/6517963 (PMC9902149; doi:10.1155/2023/6517963)
Supplement: Supplementary Materials — Figure S1: the efficacy of AFP for prognostic prediction in patients with HCC. Figure S2: the differences in molecular characterization between the miR-135b-5p-high and miR-135b-5p-low groups. Table S1: study cases. Table S2: 59 consistently upregulated miRNAs and 3 consistently downregulated miRNAs in CA and AFP-high group. Table S3: prediction result of TransmiR database. Table S4: antibody panel of CyTOF. Table S5: antibody panel of IMC. [file 6517963.f1.zip › supplemental figure 1 legend.doc]

Figure S1: The efficacy of AFP for prognostic prediction in patients with HCC. (a) Bar plots show the association between the miR-135b-5p expression and pathological parameters in patients with HCC. (b) The Kaplan-Meier curves show the OS of patients, characterized by low or high AFP level in different cut-off criteria. VI, vascular invasion.
