# Supplementary material for: Whole-Transcriptome Sequencing Combined with High-Dimensional Proteomic Technologies Reveals the Potential Value of miR-135b-5p as a Biomarker for Hepatocellular Carcinoma
Source: Biomed Res Int. 2023 Jan 30;2023:6517963. doi: 10.1155/2023/6517963 (PMC9902149; doi:10.1155/2023/6517963)
Supplement: Supplementary Materials — Figure S1: the efficacy of AFP for prognostic prediction in patients with HCC. Figure S2: the differences in molecular characterization between the miR-135b-5p-high and miR-135b-5p-low groups. Table S1: study cases. Table S2: 59 consistently upregulated miRNAs and 3 consistently downregulated miRNAs in CA and AFP-high group. Table S3: prediction result of TransmiR database. Table S4: antibody panel of CyTOF. Table S5: antibody panel of IMC. [file 6517963.f1.zip › supplemental figure 2 legend.doc]

Figure S2: The differences in molecular characterization between the miR-135b-5p-high and -low group. (a) Heatmap shows the expression of S1, S2, and S3 subclasses genes signatures in the miR-135b-5p-high and -low groups. (b) Box plots show the expression of hepatocyte function-related genes between the miR-135b-5p-high and -low groups. (c) GSEA shows the gene sets enriched in the miR-135b-5p-high group. (d) GSEA shows the gene sets enriched in the miR-135b-5p-low group. **P*< 0.05; ***P*< 0.01.
