# Supplementary material for: Whole-Transcriptome Sequencing Combined with High-Dimensional Proteomic Technologies Reveals the Potential Value of miR-135b-5p as a Biomarker for Hepatocellular Carcinoma
Source: Biomed Res Int. 2023 Jan 30;2023:6517963. doi: 10.1155/2023/6517963 (PMC9902149; doi:10.1155/2023/6517963)
Supplement: Supplementary Materials — Figure S1: the efficacy of AFP for prognostic prediction in patients with HCC. Figure S2: the differences in molecular characterization between the miR-135b-5p-high and miR-135b-5p-low groups. Table S1: study cases. Table S2: 59 consistently upregulated miRNAs and 3 consistently downregulated miRNAs in CA and AFP-high group. Table S3: prediction result of TransmiR database. Table S4: antibody panel of CyTOF. Table S5: antibody panel of IMC. [file 6517963.f1.zip › supplemental Table1.doc]

**Table S1 Study cases.**

| **ID** | **Serum AFP level (ng/ml)** | **RNA-seq** | **MicroRNA-seq** | **Mass cytometry** | **Imaging mass cytometry** |
| --- | --- | --- | --- | --- | --- |
| Sample 1 | 3.40 | Yes | Yes | Yes | Yes |
| Sample 2 | 4.70 | Yes | Yes | Yes | Yes |
| Sample 3 | 3.68 | Yes | Yes | Yes | NA |
| Sample 4 | 26.75 | Yes | Yes | NA | NA |
| Sample 5 | ＞2000.00 | Yes | Yes | Yes | Yes |
| Sample 6 | ＞2000.00 | Yes | Yes | Yes | NA |
| Sample 7 | ＞2000.00 | Yes | Yes | NA | NA |
| Sample 8 | ＞2000.00 | Yes | Yes | Yes | NA |
| Sample 9 | 5.14 | Yes | Yes | Yes | Yes |
| Sample 10 | ＞2000.00 | Yes | Yes | Yes | Yes |
| Sample 11 | 1.52 | Yes | Yes | Yes | Yes |
| Sample 12 | ＞2000.00 | Yes | Yes | NA | Yes |
| Sample 13 | 10.23 | Yes | Yes | NA | Yes |
| Sample 14 | ＞2000.00 | Yes | Yes | Yes | Yes |
| Sample 15 | 32.58 | Yes | Yes | Yes | Yes |
| Sample 16 | ＞2000.00 | Yes | Yes | Yes | Yes |
| Sample 17 | ＞2000.00 | Yes | Yes | Yes | Yes |
| Sample 18 | ＞2000.00 | Yes | Yes | Yes | Yes |
| Sample 19 | ＞2000.00 | Yes | Yes | Yes | Yes |
| Sample 20 | 19.20 | Yes | Yes | NA | Yes |
| Sample 21 | 2.17 | Yes | Yes | Yes | NA |
| Sample 22 | 1.44 | Yes | Yes | Yes | NA |
| Sample 23 | 4.98 | Yes | Yes | Yes | Yes |
| Sample 24 | 53.46 | Yes | Yes | NA | NA |
| Sample 25 | 16.40 | Yes | Yes | NA | Yes |
| Sample 26 | 1467.36 | Yes | Yes | NA | Yes |
| Sample 27 | 49.09 | Yes | Yes | NA | Yes |
| Sample 28 | >2000.00 | Yes | Yes | NA | Yes |
| Sample 29 | 166.75 | Yes | Yes | Yes | Yes |
| Sample 30 | >2000 | Yes | Yes | NA | Yes |
| Sample 31 | 56.82 | Yes | Yes | NA | Yes |
| Sample 32 | 95.94 | Yes | Yes | Yes | NA |
| Sample 33 | >2000.00 | Yes | Yes | Yes | Yes |
| Sample 34 | 319.79 | Yes | Yes | NA | NA |
| Sample 35 | 21.93 | Yes | Yes | Yes | NA |
| Sample 36 | >2000.00 | Yes | Yes | Yes | Yes |
| Sample 37 | 282.61 | Yes | Yes | NA | Yes |
| Sample 38 | 126.87 | Yes | Yes | NA | Yes |
| Sample 39 | 12.60 | Yes | Yes | Yes | NA |
| Sample 40 | 3.63 | Yes | Yes | Yes | Yes |
| Sample 41 | 2.53 | Yes | Yes | Yes | Yes |
| Sample 42 | 2.51 | Yes | Yes | Yes | Yes |
| Sample 43 | 3.66 | Yes | Yes | Yes | Yes |
| Sample 44 | 60.49 | Yes | Yes | Yes | NA |
| Sample 45 | 21.78 | Yes | Yes | Yes | Yes |
| Sample 46 | 4.65 | Yes | Yes | Yes | Yes |
| Sample 47 | >2000.00 | Yes | Yes | Yes | Yes |
| Sample 48 | 28.02 | Yes | Yes | Yes | Yes |
| Sample 49 | 9.43 | Yes | Yes | Yes | Yes |
| Sample 50 | 7.15 | Yes | Yes | NA | Yes |
| Sample 51 | 62.18 | Yes | Yes | Yes | NA |
| Sample 52 | 4.74 | Yes | Yes | Yes | Yes |
| Sample 53 | 2.13 | Yes | Yes | Yes | NA |
| Sample 54 | 5.27 | Yes | Yes | Yes | Yes |
| Sample 55 | >2000.00 | Yes | Yes | Yes | NA |
| Sample 56 | 33.53 | Yes | Yes | Yes | Yes |
| Sample 57 | >2000.00 | Yes | Yes | Yes | Yes |
| Sample 58 | 209.88 | Yes | Yes | Yes | Yes |
| Sample 59 | >2000.00 | Yes | Yes | Yes | Yes |
| Sample 60 | 9.96 | Yes | Yes | Yes | Yes |
| Sample 61 | >2000.00 | Yes | Yes | Yes | Yes |
| Sample 62 | >2000.00 | Yes | Yes | Yes | Yes |
| Sample 63 | 47.72 | Yes | Yes | Yes | NA |
| Sample 64 | 278.98 | Yes | Yes | NA | NA |
| Sample 65 | 4.71 | Yes | Yes | Yes | Yes |
