# Supplementary material for: Whole-Transcriptome Sequencing Combined with High-Dimensional Proteomic Technologies Reveals the Potential Value of miR-135b-5p as a Biomarker for Hepatocellular Carcinoma
Source: Biomed Res Int. 2023 Jan 30;2023:6517963. doi: 10.1155/2023/6517963 (PMC9902149; doi:10.1155/2023/6517963)
Supplement: Supplementary Materials — Figure S1: the efficacy of AFP for prognostic prediction in patients with HCC. Figure S2: the differences in molecular characterization between the miR-135b-5p-high and miR-135b-5p-low groups. Table S1: study cases. Table S2: 59 consistently upregulated miRNAs and 3 consistently downregulated miRNAs in CA and AFP-high group. Table S3: prediction result of TransmiR database. Table S4: antibody panel of CyTOF. Table S5: antibody panel of IMC. [file 6517963.f1.zip › supplemental Table2.doc]

**Table S2 59 consistently upregulated miRNAs and 3 consistently downregulated miRNAs in CA and AFP-high group.**

| **DEmiRNAs** | **Condition** | **Log2FC between the AFP high and low groups** | **Log2FC between the CA and CP** |
| --- | --- | --- | --- |
| hsa-miR-1197 | up regulated | 5.894926688 | 6.212688719 |
| hsa-miR-1283 | up regulated | 5.437863319 | 6.993510531 |
| hsa-miR-1323 | up regulated | 6.78932336 | 6.752240408 |
| hsa-miR-135b-5p | up regulated | 1.538658683 | 2.200409686 |
| hsa-miR-154-3p | up regulated | 3.695954609 | 5.475763665 |
| hsa-miR-17-5p | up regulated | 1.006749149 | 1.084426007 |
| hsa-miR-18a-5p | up regulated | 1.955560328 | 1.971572638 |
| hsa-miR-196a-5p | up regulated | 2.535792605 | 3.541461587 |
| hsa-miR-19a-3p | up regulated | 1.162317069 | 1.534024427 |
| hsa-miR-301a-3p | up regulated | 1.832647897 | 1.32566564 |
| hsa-miR-301a-5p | up regulated | 1.955353179 | 1.237758054 |
| hsa-miR-3200-3p | up regulated | 2.703277825 | 1.554917498 |
| hsa-miR-323a-3p | up regulated | 1.855992153 | 5.223774434 |
| hsa-miR-323b-3p | up regulated | 2.659529275 | 4.60644068 |
| hsa-miR-369-3p | up regulated | 1.0059374 | 3.939220013 |
| hsa-miR-373-3p | up regulated | 9.563013786 | 6.537028629 |
| hsa-miR-380-3p | up regulated | 5.07421194 | 5.00983131 |
| hsa-miR-382-3p | up regulated | 1.011954095 | 1.550776659 |
| hsa-miR-409-5p | up regulated | 1.066745066 | 3.695797906 |
| hsa-miR-412-5p | up regulated | 3.101212863 | 4.749693789 |
| hsa-miR-431-5p | up regulated | 1.625805549 | 4.722796082 |
| hsa-miR-483-3p | up regulated | 1.947500833 | 3.110492683 |
| hsa-miR-483-5p | up regulated | 1.384402435 | 3.030085946 |
| hsa-miR-493-3p | up regulated | 1.435525268 | 4.125362702 |
| hsa-miR-493-5p | up regulated | 1.209305108 | 4.279137591 |
| hsa-miR-498 | up regulated | 11.67637988 | 6.247769428 |
| hsa-miR-512-5p | up regulated | 9.989984063 | 6.822333607 |
| hsa-miR-515-3p | up regulated | 10.4842805 | 7.13451681 |
| hsa-miR-515-5p | up regulated | 7.521415433 | 7.042348775 |
| hsa-miR-516a-5p | up regulated | 7.54815027 | 7.842459078 |
| hsa-miR-516b-5p | up regulated | 7.279608063 | 6.807965979 |
| hsa-miR-517b-3p | up regulated | 7.925705749 | 6.375175027 |
| hsa-miR-517c-3p | up regulated | 7.078359254 | 6.500691475 |
| hsa-miR-518b | up regulated | 8.075222821 | 6.705960181 |
| hsa-miR-518c-3p | up regulated | 7.210100459 | 6.75330562 |
| hsa-miR-518d-3p | up regulated | 9.576707911 | 6.617304929 |
| hsa-miR-518e-3p | up regulated | 10.00873042 | 6.660230381 |
| hsa-miR-518e-5p | up regulated | 7.281369531 | 6.992395877 |
| hsa-miR-518f-3p | up regulated | 7.388982605 | 6.54917456 |
| hsa-miR-519a-3p | up regulated | 7.549403712 | 8.245826462 |
| hsa-miR-519b-3p | up regulated | 8.793717351 | 6.571266887 |
| hsa-miR-519c-3p | up regulated | 7.294228019 | 6.669571548 |
| hsa-miR-519d-3p | up regulated | 7.735747019 | 6.713995652 |
| hsa-miR-519e-5p | up regulated | 10.46104656 | 8.491565313 |
| hsa-miR-520a-3p | up regulated | 9.010634617 | 9.848631669 |
| hsa-miR-520a-5p | up regulated | 6.726119522 | 6.623018376 |
| hsa-miR-520b | up regulated | 24.1331544 | 6.949876397 |
| hsa-miR-520c-5p | up regulated | 25.22228858 | 6.609220567 |
| hsa-miR-520d-3p | up regulated | 3.717692086 | 4.864080759 |
| hsa-miR-520d-5p | up regulated | 9.046117856 | 7.305301897 |
| hsa-miR-520g-5p | up regulated | 6.070929275 | 6.178475686 |
| hsa-miR-524-5p | up regulated | 7.216123903 | 7.01002319 |
| hsa-miR-525-3p | up regulated | 24.39843896 | 6.607422442 |
| hsa-miR-526b-3p | up regulated | 24.45684173 | 7.0217494 |
| hsa-miR-526b-5p | up regulated | 6.033468413 | 6.571165289 |
| hsa-miR-539-3p | up regulated | 1.030024607 | 3.676053538 |
| hsa-miR-6844 | up regulated | 1.287251137 | 1.457865351 |
| hsa-miR-7-5p | up regulated | 1.698206529 | 1.201930554 |
| hsa-miR-889-3p | up regulated | 1.25328986 | 4.521275286 |
| hsa-miR-139-5p | down regulated | -2.168611078 | -1.192207995 |
| hsa-miR-378c | down regulated | -1.392966581 | -1.061429528 |
| hsa-miR-378d | down regulated | -1.251030484 | -1.211915729 |
