# Supplementary material for: Whole-Transcriptome Sequencing Combined with High-Dimensional Proteomic Technologies Reveals the Potential Value of miR-135b-5p as a Biomarker for Hepatocellular Carcinoma
Source: Biomed Res Int. 2023 Jan 30;2023:6517963. doi: 10.1155/2023/6517963 (PMC9902149; doi:10.1155/2023/6517963)
Supplement: Supplementary Materials — Figure S1: the efficacy of AFP for prognostic prediction in patients with HCC. Figure S2: the differences in molecular characterization between the miR-135b-5p-high and miR-135b-5p-low groups. Table S1: study cases. Table S2: 59 consistently upregulated miRNAs and 3 consistently downregulated miRNAs in CA and AFP-high group. Table S3: prediction result of TransmiR database. Table S4: antibody panel of CyTOF. Table S5: antibody panel of IMC. [file 6517963.f1.zip › supplemental Table3.doc]

**Table S3 Prediction result of TransmiR database.**

| **TF name:** | **SOX9** |  |  |  |  |  |
| --- | --- | --- | --- | --- | --- | --- |
| miRNA name | TSS | Binding site | Action type | Evidence | Score | Zscore |
| hsa-mir-135b | chr1: 205456086(NR_103783) | chr1: 205455656-205455670(+) | Regulation | level 1 | 853 | 1.68 |
| hsa-mir-135b | chr1: 205456086(NR_103783) | chr1: 205455657-205455672(+) | Regulation | level 1 | 811 | 1.71 |
| hsa-mir-135b | chr1: 205456086(NR_103783) | chr1: 205455706-205455724(+) | Regulation | level 1 | 787 | 1.64 |
