# Supplementary material for: Whole-Transcriptome Sequencing Combined with High-Dimensional Proteomic Technologies Reveals the Potential Value of miR-135b-5p as a Biomarker for Hepatocellular Carcinoma
Source: Biomed Res Int. 2023 Jan 30;2023:6517963. doi: 10.1155/2023/6517963 (PMC9902149; doi:10.1155/2023/6517963)
Supplement: Supplementary Materials — Figure S1: the efficacy of AFP for prognostic prediction in patients with HCC. Figure S2: the differences in molecular characterization between the miR-135b-5p-high and miR-135b-5p-low groups. Table S1: study cases. Table S2: 59 consistently upregulated miRNAs and 3 consistently downregulated miRNAs in CA and AFP-high group. Table S3: prediction result of TransmiR database. Table S4: antibody panel of CyTOF. Table S5: antibody panel of IMC. [file 6517963.f1.zip › supplemental Table4.doc]

**Table S4 Antibody panel of CyTOF.**

| **Antibody** | **Metal** | **Clone** | **Source** |
| --- | --- | --- | --- |
| AFP | 150Nd | 189506 | Fluidigm |
| ALDH | 147Sm | 44/ALDH | Fluidigm |
| CD13 | 160Gd | RV202 | Fluidigm |
| CD24 | 166Er | ML5 | Fluidigm |
| CD34 | 149Sm | 581 | Fluidigm |
| CD44 | 171Yb | IM7 | Fluidigm |
| CD45 | 89Y | HI30 | Fluidigm |
| CD47 | 209Bi | CC2C6 | Fluidigm |
| CD54 | 170Er | HA58 | Fluidigm |
| CD90 | 158Gd | 5E10 | Biolegend |
| CD104 | 173Yb | 58XB4 | Fluidigm |
| CD133 | 153Eu | 170411 | R&D |
| CD166 | 145Nd | 3A6 | Biolegend |
| CD274 | 175Lu | 29E.2A3 | Fluidigm |
| CD325 | 148Nd | 8C11 | Biolegend |
| CD326 | 141Pr | 9C4 | Fluidigm |
| CK19 | 162Dy | A53-B/A2 | Biolegend |
| c_Myc | 176Yb | 9E10 | Fluidigm |
| DNMT3B | 164Dy | 832121 | Fluidigm |
| HepPar_1 | 151Eu | EP-2 | Novus |
| H2A.Z | 165HO | EPR6171(2)(B) | Abcam |
| LGR5 | 155Gd | SA222C5 | Biolegend |
| Met | 167Er | D1C2 | Fluidigm |
| MUC_1 | MUC1 | SM3 | Abcam |
| Nanog | 169Tm | N31-355 | Fluidigm |
| OV6 | 152Sm | OV-6 | R&D |
| p21 | 159Tb | 12D1 | Fluidigm |
| p53 | 143Nd | Do-7 | Fluidigm |
| Vimentin | 156Gd | RV202 | Fluidigm |
