# Supplementary material for: Whole-Transcriptome Sequencing Combined with High-Dimensional Proteomic Technologies Reveals the Potential Value of miR-135b-5p as a Biomarker for Hepatocellular Carcinoma
Source: Biomed Res Int. 2023 Jan 30;2023:6517963. doi: 10.1155/2023/6517963 (PMC9902149; doi:10.1155/2023/6517963)
Supplement: Supplementary Materials — Figure S1: the efficacy of AFP for prognostic prediction in patients with HCC. Figure S2: the differences in molecular characterization between the miR-135b-5p-high and miR-135b-5p-low groups. Table S1: study cases. Table S2: 59 consistently upregulated miRNAs and 3 consistently downregulated miRNAs in CA and AFP-high group. Table S3: prediction result of TransmiR database. Table S4: antibody panel of CyTOF. Table S5: antibody panel of IMC. [file 6517963.f1.zip › supplemental Table5.doc]

**Table S5 Antibody panel of IMC.**

| **Antibody** | **Metal** | **Clone** | **Source** |
| --- | --- | --- | --- |
| Argianase | 164Dy | Rabbit | NOUVS |
| Beta_Catenin | 165Ho | D13A1 | Fluidigm |
| CD3 | 170Er | Polyclonal | Fluidigm |
| CD4 | 159Tb | RPA-T4 | BioLegend |
| CD8a | 162Dy | C8/144B | Fluidigm |
| CD20 | 161Dy | H1 | Fluidigm |
| CD31 | 156Gd | JC/70A | Abcam |
| CD34 | 154Sm | Rabbit | Abcam |
| CD44 | 153Eu | Rabbit | Abcam |
| CD45 | 152Sm | CD45-2B11 | Fluidigm |
| CD66a | 171Yb | CD66a-B1.1 | Fluidigm |
| CD68 | 141Pr | KPI | Abcam |
| CD90 | 163Dy | 7E1B11 | Abcam |
| CD133 | 172Yb | Rabbit | Abcam |
| CD326 | 150Nd | Rabbit | Abcam |
| CK7 | 151Eu | RCK105 | Abcam |
| CK19 | 173Yb | A53-B/A2 | BioLegend |
| Collagen_I | 169Tm | Polyclonal | Fluidigm |
| E_Cadherin | 158Gd | 24E10 | Fluidigm |
| FAP | 147Sm | Rabbit | Abcam |
| Foxp3 | 155Gd | Rabbit | NOUVS |
| GranzymeB | 167Er | EPR20129-217 | Fluidigm |
| HepPar_1 | 176Yb | OCH1E5 | Abcam |
| Ki67 | 175Lu | Rabbit | Abcam |
| Pan_CK | 160Gd | c-11 | Abcam |
| PD_L1 | 145Nd | 73-10 | Abcam |
| Vimentin | 143Nd | RV202 | Fluidigm |
| YAP1 | 149Sm | Mouse | Abcam |
| α-SMA | 142Nd | Polyclonal | Abcam |
